# Commentary: Profiles of paediatric patients experiencing stroke-like episodes associated with mitochondrial disease

**DOI:** 10.3389/fneur.2026.1775667

**Published:** 2026-04-15

**Authors:** Josef Finsterer

**Affiliations:** Neurology Department, Neurology and Neurophysiology Center, Vienna, Austria

**Keywords:** L-arginine, MELAS, mitochondrial disorder, seizure, stroke-like episode

We read with interest the article by Molla et al. on a retrospective, multicenter, observational cohort study of clinical, radiological, EEG, and genetic findings in pediatric patients with a mitochondrial disorder (MID) who also suffered a stroke-like episode (SLE) (1). It has been found that SLEs are most common in patients with MELAS and those with POLG1 variants, that EEG can help in the identification of SLEs, and that the clinical presentation of SLEs can be improved by L-arginine (1). The study is interesting, but some points should be discussed.

The first point concerns the retrospective design of the study which have disadvantages as they rely on the analysis of medical records that were not originally intended for research purposes (2).

The second point is that no definition of SLE was provided (1). Were SLEs diagnosed in the presence of specific clinical symptoms or specific imaging abnormalities known as stroke-like lesions (SLLs) on multimodal MRI? SLLs are characterized by T2/FLAIR, PWI, and DWI hyperintensity, OEF hypointensity, expansion to a nadir, and subsequent regression until the lesion completely resolves or remains as a white matter lesion, cyst, laminar cortical necrosis, focal atrophy, or toenail sign (3). Typically, SLLs are not confined to a vascular territory.

The third point is that the MRI findings of the 13 patients with SLE were not described in detail (1). Since MRI showed a cerebral lesion in only 7/13 patients with a SLE, we should know at what point in time in relation to SLE the MRI examinations were performed. In particular, for the six patients with SLE but without lesions on MRI, it should be specified how long after the onset of the SLE the MRI examinations were performed. It should also be specified whether the lesions found in the 7 patients with SLE were acute or residual from a previous SLE or had other causes. Were MRI scans performed on all 13 patients when SLE occurred?

The fourth point refers to Table 5, which shows that brain lesions regressed in 8 of 15 patients within a period of 2 to >12 months (1). Did all of these patients truly have a SLE, or did lesions other than those attributable to SLE also regress?

The fifth point is that it remains unclear why nine patients were diagnosed with MELAS even though they did not have a SLE, as shown in Figure 1 (1). Since MELAS is characterized by SLEs and other organ manifestations (4, 5), it should be clarified according to which diagnostic criteria MELAS was diagnosed in these patients.

The sixth point concerns the discrepancy between the title “Profiles of pediatric patients with…” and Table 1, which shows that 6 of the 34 patients included were over 20 years of age (1). This discrepancy should be resolved. There is also a discrepancy between the title “Patients with SLE…” and the inclusion of 21 patients who did not have SLE. The objectives of the study should be consistent with the title.

Overall, before diagnosing SLEs, the definition used to identify them should be presented. It should also be specified at what point in time cerebral imaging was performed in patients with a SLE.
